# Successful Surgical Intervention for Purulent Pericarditis Caused by Klebsiella pneumoniae Perforating Into the Thoracic Cavity: A Case Report

**DOI:** 10.7759/cureus.79625

**Published:** 2025-02-25

**Authors:** Toshinari Ito, Yoshimasa Akiba, Saki Ishiya, Toshiki Okasaka

**Affiliations:** 1 Thoracic Surgery, Toyota Kosei Hospital, Toyota, JPN

**Keywords:** empyema, klebsiella pneumoniae, perforated thoracic cavity, purulent pericarditis, video-assisted thoracic surgery

## Abstract

Purulent pericarditis is an infection of the pericardial space surrounding the heart especially caused by bacteria. An 85-year-old woman was brought to the emergency room with sudden chest pain. Chest x-ray showed cardiac hypertrophy, and a computed tomography (CT) scan revealed an air-fluid level in her pericardium and a small amount of left pleural effusion. CT scan on the day after admission showed a marked increase in left pleural effusion. The left intercostal drain yielded *Klebsiella pneumoniae* and based on these findings, surgery including pericardial and intrathoracic curettage was contemplated. Intraoperative findings showed communication between the pericardial space and the thoracic cavity. After curettage and irrigation of the pericardial space and thoracic cavity, the pericardial space was opened, and the surgery was completed. The patient’s postoperative course was uneventful. Thereafter, no recurrence or exacerbation was observed. Early pericardial and thoracic drainage followed by surgical treatment were deemed crucial in saving the patient's life.

## Introduction

The etiology of acute pericarditis is broadly classified into infectious and noninfectious, and infectious causes are further classified into bacterial, fungal, and viral. Pericarditis caused by bacteria is called purulent pericarditis, which is a rare disease that accounts for less than 1% of all pericarditis [1,2]. Purulent pericarditis has become a rare disease since the introduction of antibiotics, but if it occurs, mortality rates are reported to be 80% without appropriate treatment and 40% with appropriate treatment [3]. Early action is essential because of the high mortality rate without appropriate treatment.

Its treatment primarily recommends intravenous antibiotic administration and effective pericardial drainage. Antibiotics are typically administered after blood cultures are obtained, starting with broad-spectrum antibiotics and later changing to narrow-spectrum based on sensitivity results. However, if inadequate, subxiphoid pericardiotomy, rinsing of the pericardial cavity, and intrapericardial thrombolysis should be considered. Pericardiectomy should also be considered for thick adhesions, localized or thick purulent pericardial effusions, recurrent tamponade, persistent infection, and progression to stenosis [2,4]. Here, we report a case of purulent pericarditis and intrathoracic perforation, an extremely rare condition in which antibiotic therapy and surgical treatment, including pericardial and intrathoracic curettage and pericardial fenestration, was lifesaving.

## Case presentation

**Figure 1:**
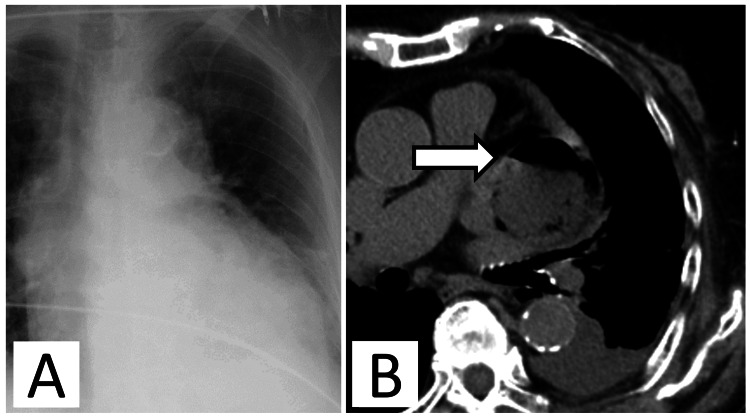
Imaging findings on the day of admission. (A) Chest x-ray showing cardiomegaly. (B) Axial chest computed tomography (CT) revealing fluid accumulation in the pericardial space; air is observed inside the liquid, and an air-fluid level is observed (white arrow).

An 85-year-old woman presented to the emergency room with sudden chest pain when visiting our outpatient clinic. On arrival at the hospital, her blood pressure was 96/58 mmHg, pulse rate was 99 beats/min, respiratory rate was 22 breaths per minute, and body temperature was 38.1℃. Two months prior, she was admitted to our hospital after complaining of chest pain and was diagnosed with idiopathic pericarditis. After oral treatment with aspirin (1.5 g per day), colchicine (0.25 mg per day), and prednisolone (15 mg per day), her condition improved, and she was discharged. Prednisolone was then tapered off. She had a history of hypertension, dyslipidemia, diabetes, and a transient ischemic attack. Chest x-ray showed cardiomegaly, and contrast-enhanced chest computed tomography (CECT) revealed a mass (50 × 55 × 80 mm) in the left dorsal pericardial space. Air was trapped inside the lesion, and an air-fluid level was observed, suggesting abscess formation (Figures 1A, 1B).

A small amount of pleural effusion was also observed in the left thoracic cavity. Previous CT taken at the time of idiopathic pericarditis did not reveal any abnormal objects such as a pericardial cyst. Subsequently, the patient was diagnosed with pericarditis; upon hospitalization, her blood pressure was 96/58 mmHg, pulse rate was 99 beats/min, and edema was observed in both lower legs. Blood biochemical tests showed a slight increase in white blood cell count (10.6×10^3^/mm^3^), a moderate increase in C-reactive protein (CRP) level (11 mg/dL), and a marked increase in HbA1c (11.3%) and glucose level (539 mg/dL). The patient was then started on antibiotic treatment with meropenem (1 g three times per day). Owing to the localization of the abscess, performing pericardial drainage was difficult.

**Figure 2:**
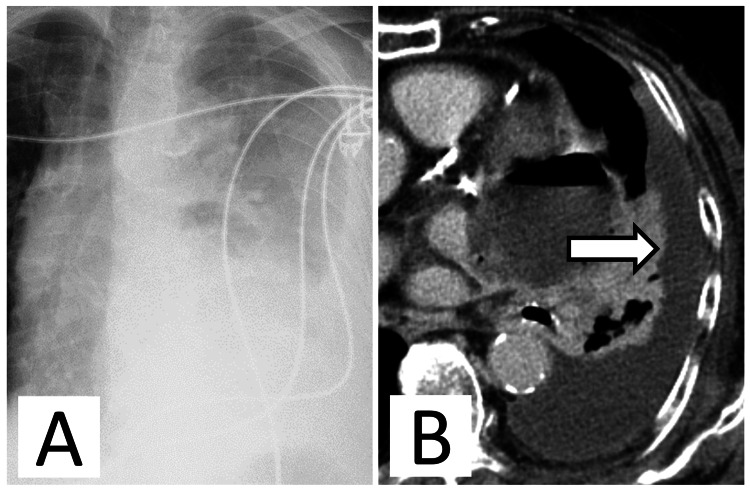
Imaging findings on the day after admission. (A) Chest x-ray showing decreased transparency in the left lung field. (B) Axial chest computed tomography revealing increased left pleural effusion (white arrow).

On the day after admission, a chest x-ray showed decreased transparency throughout the left lung field (Figure 2A), and blood tests revealed a CRP level of 28 mg/dL and increased inflammatory response. Chest CT revealed a marked increase in the left pleural effusion (Figure 2B), and when a chest tube was placed, 500 mL of purulent pleural effusion with yellowish-white turbidity was observed.

Esophagography was performed on the same day; however, no communication between the esophagus and pericardial or thoracic cavity was detected. Based on the elevated inflammatory response and a significant increase in pleural effusion, we suspected that the intrapericardial abscess was perforated into the thoracic cavity. Consequently, intrathoracic irrigation with physiological saline from a chest tube was initiated. Subsequently, *Klebsiella pneumoniae* was detected in the blood and pleural cultures, and the patient was diagnosed with purulent pericarditis, left empyema, and bacteremia. After intrathoracic lavage, echocardiography revealed that the abscess in the pericardial space persisted leading to cardiac tamponade. Chest CT showed decreased pleural effusion however, the intrapericardial abscess remained. Therefore, we decided to perform thoracoscopic pericardial fenestration and pericardial/intrathoracic curettage.

**Figure 3:**
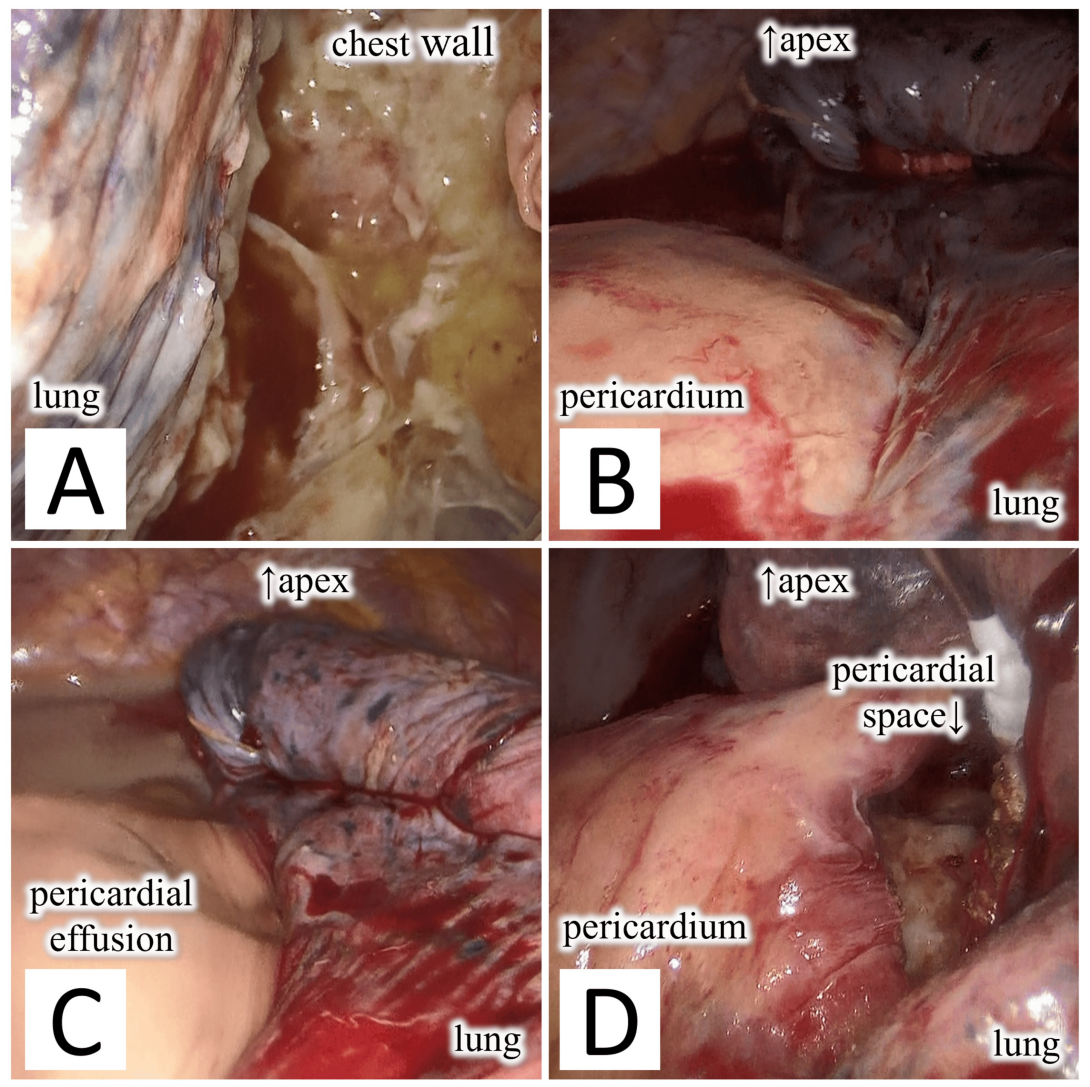
Intraoperative findings. (A) The pleura is covered with purulent membrane. (B) The pericardium is engorged and adhered to the left lung. (C) When the adhesions between the pericardial space and the left lung are manually dissected, communication between the thoracic cavity and pericardial space is observed, and purulent pericardial fluid flows out. (D) After aspiration of purulent pericardial effusion in the pericardial space, the pericardium is opened, and the pericardial space is washed.

Surgery was initiated under general anesthesia with the patient on the right lateral position, and a port was inserted into the drain insertion site at the midaxillary line of the eighth left intercostal space to observe the inside of the thoracic cavity. Inside the thoracic cavity, a purulent membrane was observed throughout the pleura, and membranous adhesions and multiloculated yellow transparent pleural effusions were observed on the dorsal side (Figure 3A). Next, a 4-cm incision was made in front of the fifth intercostal space, and a 1-cm incision was made at the inferior angle of the scapula in the sixth intercostal space to commence dissection of the lung adhesions. Upon examination, the pericardium was engorged and swollen, and part of the inferior lingular segment of the lung adhered to the pericardium (Figure 3B). When the adhesions were dissected, there was communication with the pericardium, and purulent pericardial fluid leaked out (Figure 3C). After aspirating as much pericardial fluid as possible, the pericardium was incised, and the communication hole was opened to a diameter of 3 × 2 cm (Figure 3D).

**Figure 4:**
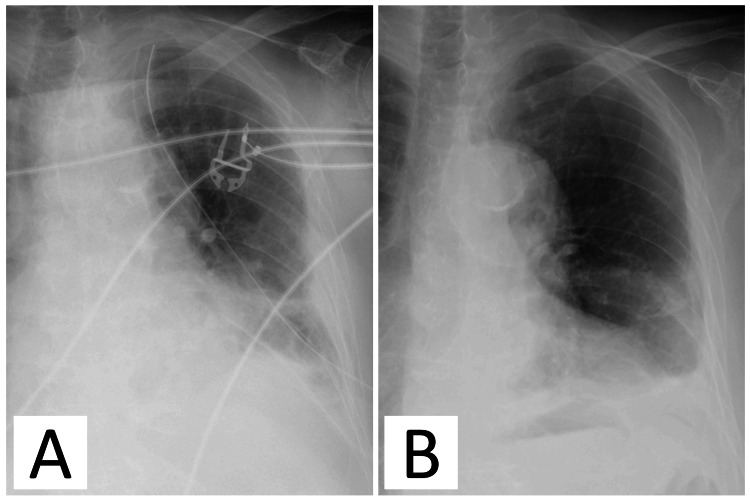
Postoperative imaging findings. (A) Chest x-ray on the day after surgery. (B). Final chest x-ray before discharge.

Subsequently, the inside of the pericardial space and thoracic cavity were curetted and washed. Drains were placed in the lung apex on the dorsal side of the thoracic cavity and inside the pericardial space, and the surgery was completed (Figure 4A).

The postoperative antibiotic was changed to cefmetazole sodium (1 g twice per day) based on susceptibility test results. After multiple confirmations that the culture test results of the drainage fluid from the pericardial drain and chest tube were negative, the pericardial drain was removed on the seventh postoperative day, and the chest tube was removed on the ninth postoperative day. Antibiotics were then switched to oral amoxicillin 500 mg/clavulanic acid 125 mg three times per day, and the patient was discharged on the 18th hospitalization day (Figure 4B). At one-year postoperatively, no recurrence was observed.

## Discussion

In our case, the patient was diagnosed with purulent pericarditis. The pathogenesis of purulent pericarditis involves the following: infections (1) spreading from infection foci into the thoracic cavity or under the diaphragm; (2) spreading from intracardiac infections, such as infective endocarditis; (3) due to chest trauma or chest surgery; and (4) due to hematogenous infection from bacteremia [4]. *Staphylococcus aureus* and *Streptococcus pneumoniae* are thought to be the most likely causative bacteria; however, in this case, *K. pneumoniae* was detected in the blood and pleural fluid cultures. *K. pneumoniae* is a resident bacterium in the oral cavity and intestinal tract and is known to cause opportunistic infections owing to community-acquired infections and nosocomial infections. Purulent pericarditis caused by the same bacterium has been reported in patients undergoing hemodialysis [5], with a history of diabetes [6], and with cases secondary to diaphragmatic abscess [7]. In this case, no history of trauma or surgery before or after the onset of symptoms and nearby infection foci were observed. Based on a history of idiopathic pericarditis, the cause of the disease was thought to be the patient’s susceptibility to infection due to poorly controlled diabetes and long-term steroid therapy. The presence of bacteremia suggested a hematogenous infection in the pericardial space.

The treatment of purulent pericarditis mainly involves the administration of antibiotics and drainage, such as percutaneous pericardial drainage and pericardial fenestration. Percutaneous pericardial drainage is often performed in cases of mild invasion; however, cases in which insufficient drainage develops into constrictive pericarditis or persistent purulent pericarditis have been reported. If no improvement is observed, prompt surgical intervention is recommended [8,9]. Pericardiotomy is the method recommended in the European Society of Cardiology guidelines [1], since it is associated with a higher success rate and a lower incidence of constrictive pericarditis. A pericardiectomy is associated with a mortality of 8% but it is the approach that resolves all situations, even the most complicated (adhesions, loculated effusions, or persistent infection). In our case, percutaneous pericardial drainage was difficult because of the localization of the abscess. Because the abscess caused by pericarditis had perforated the thoracic cavity, an attempt was made to drain it into the pericardial space via thoracic drainage; however, this attempt was unsuccessful. As early surgical intervention is considered necessary when percutaneous drainage is impossible or insufficient, we decided to perform pericardial fenestration.

In addition to the transthoracic approach, options for pericardial fenestration include the midline sternotomy and subxiphoid approaches. In recent years, the median sternotomy approach has rarely been the first choice because of the degree of invasion and risk of sternal infection [10]. The subxiphoid approach although with limited access does extensive pericardial resection and prevents the spread of infection into the thoracic cavity but pericardial drainage of the dorsal surface remains a difficult option [11]. The use of a thoracoscope in the transthoracic approach is considered less invasive than median sternotomy and provides a better field of view than a subxiphoid incision [12]. While the subxiphoid approach is generally the first choice for treating purulent pericarditis to avoid the spread of infection, a left empyema was already present in our case. Therefore, after adequately washing the thoracic cavity, surgical field, and invasion site, a transthoracic approach was considered the best option.

In our case, although antibiotic treatment was initiated on the day of admission, marked increases in the left pleural effusion and inflammatory response were observed the next day. Subsequently, a 20 Fr chest tube was placed. However, the intrapericardial abscess persisted, and surgery was performed using a thoracoscopic transthoracic approach with three incisions. The intraoperative findings showed that the pericardial space and lungs were adherent, and when they were manually separated, the pericardial space and thoracic cavity easily communicated. Based on the course and intraoperative findings, the purulent pericarditis was deemed to have perforated into the thoracic cavity, causing empyema. The perforated site subsequently adhered to the lungs and closed due to inflammation. While reports exist of pericarditis-induced intracardiac perforation [13] or inflammation spreading from empyema to result in purulent pericarditis, to the best of our knowledge, there have been no reported cases of Purulent pericarditis-induced intrathoracic perforation accompanied by empyema. In other cardiac diseases, perforation into the chest cavity from cardiac tamponade is not common and is considered very rare, and in this case, the severe inflammation caused by *K. pneumoniae* is thought to have led to perforation of the pericardium.

## Conclusions

Purulent pericarditis is rare and difficult to diagnose. Purulent pericarditis can cause serious complications, such as intracardiac perforation and infective endocarditis. This is a rare case in which the thoracic cavity was directly perforated, and empyema was present. The strategies adopted to achieve complete drainage of the pericardial space include pericardiocentesis, pericardiotomy, and pericardiectomy. Aggressive source control, such as pericardiocentesis and surgical drainage, should be performed immediately to improve clinical outcomes. If percutaneous pericardial drainage is difficult or poor drainage of the pericardial space, early surgical treatment is required to save the patient's life.
